# Kidney transplantation with preformed diabetic nephropathy kidney: review of pathological changes and clinical outcomes

**DOI:** 10.3389/fendo.2025.1599660

**Published:** 2025-07-01

**Authors:** Shujing Li, Yang Wang, Beike Chen, Maozhi Tang, Keqin Zhang, Linguo Shen

**Affiliations:** ^1^ Department of Urinary Nephropathy Center, The Second Affiliated Hospital of Chongqing Medical University, Chongqing, China; ^2^ Department of Clinical Laboratory, The First Naval Hospital of Southern Theater Command, Zhanjiang, China

**Keywords:** diabetic nephropathy, preformed diabetic nephropathy, donor, transplantation, post-transplant hyperglycemia

## Abstract

**Introduction:**

Kidney transplantation from expanded-criteria donors represents an effective approach to alleviate organ shortages. The feasibility for transplantation of donor kidneys with preformed diabetic nephropathy (DN) has not been extensively investigated.

**Search strategy:**

We performed a literature review to explore the pathological changes and clinical outcomes of kidney transplantation using preformed DN kidney. A systematic and comprehensive search was conducted from the inception to June 13, 2024.

**Results:**

Data from eight articles encompassing 103 cases were included for analysis. The pooled proportions of stable, progressive, and reversed DN-related pathological change were 0.66 (95% CI 0.56–0.77, I^2^ = 21.77%), 0.27 (95% CI 0.18–0.36, I^2^ = 10.04%) and 0.05 (95% CI 0.01–0.10, I^2^ = 0.00%), respectively. Eight-six cases were divided into post-transplant hyperglycemia group and normal post-transplant blood glucose group to evaluate the effect of post-transplant hyperglycemia on DN pathology, indicating the normal post-transplant blood glucose group had higher proportions of stable and reversed pathological states. Most cases achieved a graft survival rate of more than 80% at around five years post-transplant.

**Conclusion:**

A majority of transplantations use donor kidneys with preformed DN exhibit acceptable renal pathological changes and graft survival. However, post-transplant hyperglycemia may adversely affect the pathological progression of the kidneys, particularly in cases with long-term follow-up.

## 1 Introduction

Kidney transplantation, an optimal treatment for end-stage renal disease, is impeded by organ scarcity. A highly frustrating yet effective strategy is to enlarge the donor pool by expanding the donor criteria and fully exploiting all accessible grafts. Prior to kidney transplantation, multiple reporting systems such as the Remuzzi score, Banff criteria, and the kidney donor profile index are employed to evaluate the quality of donor kidneys; pathological features (including the rate of glomerulosclerosis, tubular atrophy, and interstitial fibrosis) assume a crucial role (1, 2).

China has one of the highest incidence rates of diabetes mellitus (DM) worldwide, and diabetic nephropathy (DN) has emerged as the primary cause of end-stage renal disease (3, 4). A considerable proportion of organ donation volunteers are patients with DM or DN, and between 2008 and 2019, half of the kidneys from donors with diabetes were rejected because of pathological lesions (5). Nevertheless, some recent large-scale retrospective studies have indicated that kidney transplants from donors with DM can effectively reduce the waiting time for recipients, and no significant differences in the glomerular filtration rate (eGFR) or urinary protein excretion have been observed compared with transplants from donors without DM (6).

Hyperglycemia induced by DM results in mesangial cell proliferation, podocyte damage, and endothelial cell decompensation, ultimately giving rise to DN (7). Glomerular lesions in DN can be categorized into four types. The diagnosis of class IV requires a ratio of over 50% of glomerular sclerosis and such tissues are evidently not suitable for transplantation. In the classification of glomerular lesions, the proportion of glomerular sclerosis below stage III has not been stated (8). Research by Mohan indicated that the DN kidney can be utilized for transplantation when the number of sclerotic glomeruli is controlled within a certain range (9). It thus appears that lesions related to DN have not been included in the criteria for rejection. Few studies have reported the feasibility of DN kidneys for transplantation, an issue that requires further exploration. Based on this predicament, a systematic review of the literature was carried out to explore the current status of clinical practice of preformed DN in kidney transplantation.

## 2 Methods

### 2.1 Search strategy and study selection

We performed a systematic and comprehensive search of the PubMed database, using kidney transplant recipient, kidney transplantation, renal transplantation, diabetic donor, diabetic nephropathy, diabetic kidney disease, diabetes mellitus, hemoglobin A1c, hyperglycemia, histology, pathology, and biopsy as keywords, with a deadline by June 13, 2024. The references of the selected articles were manually searched to obtain other relevant entries. The search strategy was presented in Additional file 1. Inclusion criteria: 1) The literatures related to kidney transplantation with DN kidney were selected; 2) Both baseline and follow-up biopsy information was described in detail; 3) Clinical outcomes of transplantation were reported. Exclusion criteria: 1) Non-English articles, conference articles, and reviews, case reports (<3 cases) were excluded; 2) Articles without baseline or follow-up pathological data on kidney biopsies were excluded. The flow chart of study selection was presented in **Supplementary Figure 1**. Baseline biopsy referred to time-zero graft kidney biopsy or first biopsy within 2 weeks post-transplantation. The relevant articles were included to assess the pathological changes related to DN, the effect of post-transplant hyperglycemia on pathological progression, and the progression of graft function.

### 2.2 Quality assessment

The methodological quality of included studies was evaluated using the Joanna Briggs Institute (JBI) Critical Appraisal Tools for case reports and case series. The assessment focused on eight criteria for case reports and ten criteria for case series, including clarity of patient demographics, comprehensiveness of case descriptions, diagnostic assessments, interventions, and outcome reporting. Each criterion was scored as “yes,” “no,” “unclear,” or “not applicable.” Studies were rated as “good” if they met ≥ 75% of the criteria, “fair” if they met 50–74%, and “poor” if they met < 50%. Detailed quality assessments for each study are presented in **Supplementary Table 1**.

### 2.3 Statistical analysis

The meta package of Stata/MP 18.0 was used. A proportion of the meta-analysis used the inverse variance method and a random effects model to estimate the magnitude of the effects. Heterogeneity was quantified with I^2^ and τ^2^ statistics. The outcomes of interest were treated as dichotomous variables, with their respective 95% confidence intervals (95% CI).

## 3 Results

### 3.1 Study characteristics

We analyzed data from eight studies (10–17) with a total of 103 recipients (**Table 1**). The mean follow-up time of patient biopsies ranged from 5 to 59.5 months. Blood glucose status after kidney transplantation was reported in 86 cases, of which 54 described normal blood glucose levels and 32 hyperglycemia. The demographic data of the included patients from the studies by Khan et al. (12), Truong et al., and Gilbert et al. (17) referred to the cohorts to which they belonged. Khan et al. and Gilbert et al. did not report the detailed baseline DN pathological classification, while pathological changes between baseline and endpoint biopsies were described.

**Table 1:** Included studies on preformed DN transplantation.

Study	Lee et al. (10)	Truong et al. (11)	Khan et al. (12)	Truong et al. (13)	Harada et al. (14)	Hsu et al. (15)	Comai et al. (16)	Gilbert et al. (17)
Sample size	34	11	17	17	3	5	10	6
Mean follow-up (baseline to final biopsies)	1 year	59 weeks	562 d	41 weeks	1 year	4.4 years	59.5 months	5 months
Recipient age (years)	53.82 ± 10.68	48.73 ± 9.71	NG	NG	31.33 (7–57)	40.16 (27.5–52.7)	59.9 ± 7	NG
Male recipient [n (%)]	20 (58.8)	5(45.46)	NG	NG	1 (33.33)	3 (60)	70%	NG
Recipient diabetes [n (%)]	11 (32.4)	2 (18.18)	NG	NG	0	0	0%	NG
Recipient HTN [n (%)]	NG	10(90.91)	NG	NG	NG	100%	100%	NG
Dialysis duration	2337.09 ± 1032.23d	NG	NG	3.58 ± 2.23 years	NG	6.12 (3.7–11.4) years	NG	NG
Cause of ESRD (n)	DM 10, HTN 3, GN 4, other 17	DN 1, HTN 5, DN+HTN 1, GN 2, other 2	NG	NG	GN 2, PKD 1	GN 1, Analgesic 1, SLE 1, other 2	GN 4, PKD 3, other 3	NG
Post-transplant hyperglycemia [n (%)]	11 (32)	5 (45.46)	NG	14(82.35)	0	0	0	4 (66.67)
Donor age (years)	60.38 ± 9.53	47.89 ± 6.76	NG	NG	58.33 (54–67)	43.8 (22–57)	69 ± 7.2	NG
Male donor [n (%)]	24 (70.6)	8 (72.73)	NG	NG	2 (66.67)	NG	60%	NG
Donor HTN [n (%)]	NG	8 (72.73)	NG	NG	NG	NG	NG	NG
Baseline allograft DN classification								
0 [n (%)]	5 (14.71)	0	NG	12 (70.59)	0	0	1 (10.00)	NG
I [n (%)]	17 (50.00)	0		0	2 (66.67)	0	1 (10.00)	
IIa [n (%)]	6 (17.65)	8 (72.73)		5 (29.41)	1 (33.33)	1 (20.00)	5 (50.00)	
IIb [n (%)]	2 (5.88)	2 (18.18)		0	0	2 (40.00)	2 (20.00)	
III [n (%)]	4 (11.76)	1 (9.09)		0	0	2 (40.00)	1 (10.00)	
Allograft DN classification change at endpoint								
Stable [n (%)]	26 (76.47)	7 (63.64)	10 (58.82)	12 (70.59)	0	2 (40.00)	4 (40.00)	5 (83.33)
Progressive [n (%)]	6 (17.65)	4 (36.36)	6 (35.29)	5 (29.41)	0	3 (60.00)	3 (30.00)	1 (16.67)
Reversed [n (%)]	2 (5.88)	0	1 (5.88)	0	3 (100)	0	3 (30.00)	0
Baseline allograft function eGFR (ml/min/1.73 m^2^) Scr(mg/dl)	eGFR 49.1 ± 22.5	NG	NG	NG	NG	eGFR: 65.67 (40.33–143.78)	Scr 1.6 ± 0.8, eGFR 54.8 ± 25.5	NG
Endpoint allograft function eGFR (ml/min/1.73 m^2^) Scr(mg/dl)	eGFR 51.2 ± 15.3	Scr 2.26 ± 1.47	NG	Scr 1.05 ± 0.52	NG	eGFR 16.01 (5.4–39.71)	Scr 1.7 ± 0.8, eGFR 50.7 ± 22.9	NG
Transplant outcomes	Six patients (17.65%) lost grafts after 48.5 months follow up.	Graft loss 1	Death-censored graft survival at 5 years: 87.5% in R-DM and 87.4% in R-N	NG	NG	Graft loss 1	Stable	NG

HTN, hypertension; ESRD, end-stage renal disease; DM, diabetes mellitus; DN, diabetic nephropathy; GN, glomerulonephritis; PKD, polycystic kidney disease; R-DM, diabetic recipient; R-N, non-diabetic recipient; Scr, serum creatinine; eGFR, glomerular filtration rate; SLE, systemic lupus erythematosus; Truong et al. (11) and Truong et al. (13) were conducted by the same first author but reported different cases.

### 3.2 Pathological changes of preformed DN transplantation

**Figure 1:**
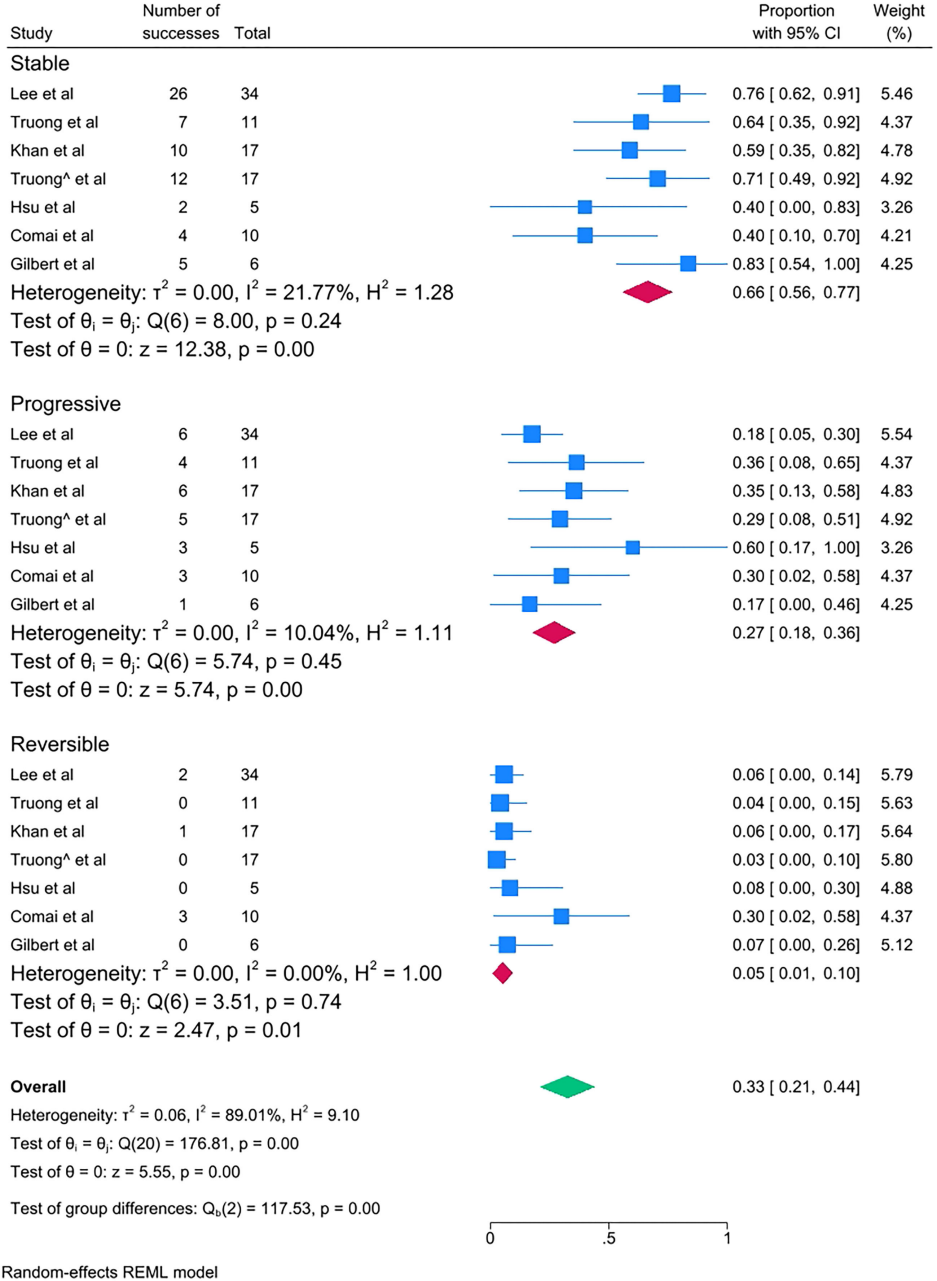
Proportional meta-analysis of DN pathological change of included cases.

The pathological changes of preformed DN transplantation were described in **Table 1**. Among the 103 recipients, proportions of stable, progressive and reversed states were 64.08% (66/103), 27.18% (28/103) and 8.74% (9/103). Proportional meta-analyses were conducted to assess the pooled proportion of stable, progressive, and reversed DN pathological changes among the examined cases. Of the 103 cases that were included in the primary meta-analysis, a high degree of heterogeneity was found in the stable (I^2^ = 64.13%) and reversible (I^2^ = 91.43%) subgroups. After excluding the study by Harada et al. (14) that reported only three cases, the degree of heterogeneity was controlled. As shown in **Figure 1**, the pooled proportion of stable DN-related pathological change was 0.66 (95% CI 0.56–0.77, I^2^ = 21.77%); the pooled proportion of progressive DN-related pathological change was 0.27 (95% CI 0.18–0.36, I^2^ = 10.04%); and the pooled proportion of reversed DN-related pathological change was 0.05 (95% CI 0.01–0.10, I^2^ = 0.00%). The above subgroup meta-analysis indicated that the degree of heterogeneity was low (I^2^<25%); therefore, no further heterogeneity analyses were performed.

### 3.3 Progression of graft function

Three studies—by Lee et al. (10), Hsu et al. (15), and Comai et al. (16)—reported that the baseline mean eGFR of graft kidney ranged from 49.1 ml/min/1.73 m^2^ to 65.67 ml/min/1.73 m^2^ after kidney transplantation, indicating relatively good primary graft function. At the endpoint (final biopsy), three studies—by Lee et al. (10), Truong et al. (13), and Comai et al. (16)—with 61 cases showed stable graft function. In contrast, two small sample-size studies—by Truong et al. (11) and Hsu et al. (15)—showed worsening graft function due to two cases of graft loss. Four studies—by Lee et al. (10), Khan et al. (12), Hsu et al. (15), and Comai et al. (16)—with mean follow-up times ranging from 48.5 months to 5 years reported long-term graft survival rates of 82.3%, 87.4–87.5%, 80%, and 100%, respectively.

### 3.4 Post-transplant hyperglycemia and pathological state of recipients

As mentioned above, post-transplant blood glucose levels were reported for 86 recipients. Among the recipients with hyperglycemia, stable pathological changes were observed in 22(25.58%), progression in 9(10.47%), and reversal in one (1.16%). In the non-hyperglycemic recipient group, DN-related changes were stable in 34(39.53%) recipients but progressed in 13(15.12%) and were reversed in seven (8.14%). Compared to hyperglycemic recipient group, the non-hyperglycemic recipient group had higher proportions of stable state (39.53% versus 25.58%) and reversed state (8.14% versus 1.16%) (**Table 2**). Of the 8 reversed cases, the pathological reversal time was about one-year post-transplant in four cases, about two years post-transplant in two cases and about five years post-transplant in two cases. To investigate the effect of the duration of post-transplant hyperglycemia on DN pathology, the 86 recipients were divided into two groups based on whether the follow-up time exceeded one year. Accordingly, 57 recipients were followed up for less than one year and 29 for more than one year. In the former group, pathological changes remained stable in 41 patients (71.93%), progressed in 10(17.54%), and were reversed in six (10.53%). Among the recipients with a follow-up time of more than one year, a stable state was reported for 15(51.72%), progression for 12(41.38%), and reversal in two (6.90%), indicating a higher proportion of progression state (41.38% versus 17.54%) when compared to recipients with a follow-up time less than one year (**Table 3**).

**Table 2:** Distribution of pathological states in post-transplant hyperglycemia and normal post-transplant blood glucose recipients.

Study (sample size)	Pathological states of recipients with post-transplant hyperglycemia			Pathological states of recipients with normal post-transplant blood glucose		
	Stable	Progressive	Reversed	Stable	Progressive	Reversed
Lee et al. (10) (34)	9 (26.47)	1 (2.94)	1 (2.94)	17 (50.00)	5 (14.71)	1 (2.94)
Truong et al. (11) (11)	3 (27.27)	2 (18.18)	0	4 (36.36)	2 (18.18)	0
Truong et al. (13) (17)	9 (52.94)	5 (29.41)	0	3 (17.65)	0	0
Harada et al. (14) (3)	0	0	0	0	0	3 (100)
Hsu et al. (15) (5)	0	0	0	2 (40.00)	3 (60.00)	0
Comai et al. (16) (10)	0	0	0	4 (40.00)	3 (30.00)	3 (30.00)
Gilbert et al. (17) (6)	1 (16.67)	1 (16.67)	0	4 (66.67)	0	0
Total(n=86)	22 (25.58)	9 (10.47)	1 (1.16)	34 (39.53)	13 (15.12)	7 (8.14)

**Table 3:** Pathological states of recipients in different follow-up periods.

Pathological state	Follow-up time	
	≤1 year (n=57)	>1 year (n=29)
Stable	41 (71.93)	15 (51.72)
*Post-transplant hyperglycemia*	13 (22.81)	9 (31.03)
*Normal post-transplant blood glucose*	28 (49.12)	6 (20.69)
Progressive	10 (17.54)	12 (41.38)
*Post-transplant hyperglycemia*	4 (7.02)	5 (17.24)
*Normal post-transplant blood glucose*	6 (10.53)	7 (24.14)
Reversed	6 (10.53)	2 (6.90)
*Post-transplant hyperglycemia*	1 (1.75)	0
*Normal post-transplant blood glucose*	5 (8.77)	2 (6.90)

## 4 Discussion

The main objective for preparing this comprehensive review was to conduct an in-depth investigation to determine whether organs with preformed DN could be deemed effective for transplantation. In 1983, a remarkable study reported that a recipient without diabetes received a kidney from a donor with confirmed DN, and a detailed graft biopsy procedure revealed a surprising reversal of DN lesions at seven months post-transplant (18, 19). These constitute the earliest reports to suggest that preformed DN can indeed be selected for transplantation and that the alterations associated with preformed DN have the potential to be reversed in conjunction with changes in the recipient’s glucose levels.

Among the included preformed DN kidney transplantations, a stable state in terms of pathological changes was noted in the majority of the recipients, progression state accounted for a lower proportion, while reversed state accounted for 8.74%. In this review, we analyzed two influence factors of DN progression including post-transplant hyperglycemia and follow-up time. With regard to the effect of post-transplant hyperglycemia, recipients without hyperglycemia had higher proportions of stable and reversed pathological change, suggesting that the post-transplant hyperglycemic state is critical for the progression of preformed DN renal pathology. Meanwhile, we found included recipients whose follow-up time less than one year had higher proportions of stable and reversed state, and had a lower proportion of progressive state. However, the proportion of progressive state increased notably in the context of follow-up time exceed one year. As we know, DN progression is influenced by a combination of multiple factors over time. Generally, early ischemia–reperfusion injury, potential acute and chronic rejection, viral infection, and extensive use of immunosuppressive drugs can have a significant impact on the pathological progression of preformed DN after renal transplantation. It is important to note that these factors interact and compound one another’s effects, creating a complex web of influences that can further complicate prognosis (20–22).

Among included recipients, a few instances were recorded in which the pathological changes were reversed after transplantation, and pathological reversal was more common in patients without hyperglycemia than in those with hyperglycemia after transplantation. Wu et al. found that early-stage DN is reversible, which is related to reduced NOX expression and improvement in mitochondrial function after transplantation (23). Previous studies on islet or pancreatic transplantation imply that pathological tissues such as in DN undergo remodeling after normal blood glucose levels are achieved, but remodeling demands a longer time, approximately 5 years (24–26). Through a comprehensive literature review of the transplantations using donor with preformed DN, we identified four cases of DN pathological reversal under the circumstances of non-hyperglycemia after a long-term follow-up, two were reversed at five-year post-transplant (16), one was reversed at seven-year post-transplant (27), and one was at nine-year post-transplant (28), respectively. The above studies reflected that donor DN pathology can be reversed at early or late post-transplant period, and normal post-transplant blood glucose is crucial for pathological reversal.

Regarding the mean baseline eGFR post-transplant, our conclusion based on the data in the included long-term follow-up studies was that the range 49.1–65.67 ml/min/1.73 m^2^ is comparable with the data presented in the 2022 OPTN/SRTR report (45 ml/min/1.73 m^2^) (29). Four studies reported long-term graft survival rates of 82.3%, 87.4–87.5%, 80%, and 100%, which are comparable with the data presented in the 2022 OPTN/SRTR report—a five-year graft survival rate of 81.4% in deceased donor kidney transplant recipients aged 18–34 years (29). These studies indicated the preformed DN transplantation can achieve comparable graft survival to that of conventional kidney transplantation.

In conclusion, kidney transplantation from donors with preformed DN presented stable pathological changes in most cases and was accompanied with acceptable graft survival. Normal post-transplant blood glucose was beneficial to DN pathological stability and pathological reversal. Unfortunately, the restricted number of cases and limited follow-up time negatively affects the validity of our conclusions, and more high-quality cohort studies are urgently needed to explore this important clinical question.
