# Supplementary material for: Kidney transplantation with preformed diabetic nephropathy kidney: review of pathological changes and clinical outcomes
Source: Front Endocrinol (Lausanne). 2025 Jul 1;16:1599660. doi: 10.3389/fendo.2025.1599660 (PMC12259419; doi:10.3389/fendo.2025.1599660)
Supplement: Supplementary file 1 [file DataSheet1.docx]

**Search strategy**

Database: Pubmed

Search from 1967/1/1 - 2024/12/1

#1: ((kidney transplant recipient (KTR[Title/Abstract])) OR (kidney transplantation [Title/Abstract])) OR (renal transplantation [Title/Abstract])

#2: (((((diabetic donor [Title/Abstract]) OR (diabetic nephropathy [Title/Abstract])) OR (diabetic kidney disease [Title/Abstract])) OR (diabetes mellitus [Title/Abstract])) OR (hemoglobin A1c[Title/Abstract])) OR (hyperglycemia [Title/Abstract])

#3: ((histolog*[Title/Abstract]) OR (patholog*[Title/Abstract])) OR (biopsy [Title/Abstract])

#4: #1 and #2 and #3 138 literatures are identified

**Supplementary Table 1: Quality assessment of included studies using JBI**

| **Study ID** | **Q1** | **Q2** | **Q3** | **Q4** | **Q5** | **Q6** | **Q7** | **Q8** | **Overall appraisal** |
| --- | --- | --- | --- | --- | --- | --- | --- | --- | --- |
| Lee et al. (10) | Yes | Yes | Yes | Yes | Yes | No | No | Yes | Good |
| Truong et al. (11) | Yes | Yes | Yes | Yes | Yes | Yes | No | Yes | Good |
| Khan et al. (12) | Yes | Yes | Yes | Yes | Yes | No | No | Yes | Good |
| Truong et al. (13) | Yes | Yes | Yes | Yes | Yes | No | No | Yes | Good |
| Harada et al. (14) | Yes | Yes | Yes | Yes | Yes | Yes | No | Yes | Good |
| Hsu et al. (15) | Yes | Yes | Yes | Yes | Yes | Yes | No | Yes | Good |
| Comai et al. (16) | Yes | Yes | Yes | Yes | Yes | Yes | No | Yes | Good |
| Gilbert et al. (17) | Yes | Yes | Yes | Yes | Yes | Yes | No | Yes | Good |

**Supplementary Figure 1 Flow chart of study selection**

*138 literatures from PubMed*

**Identification**

*23 excluded*

*Review: 11*

*Case report (sample size<3): 6*

*No baseline of follow data of biopsy: 6*

*107 excluded via titles and abstracts screening*

**Included**

*8 studies included*

**Screening**

*31 relevant*
